# Breaking the silos, stopping the spread: an interview with Jyoti Joshi

**DOI:** 10.1242/dmm.049626

**Published:** 2022-05-20

**Authors:** Jyoti Joshi

**Affiliations:** Department of Science, International Centre for Antimicrobial Resistance Solutions, Ørestads Boulevard 5, 2300 Copenhagen, Denmark


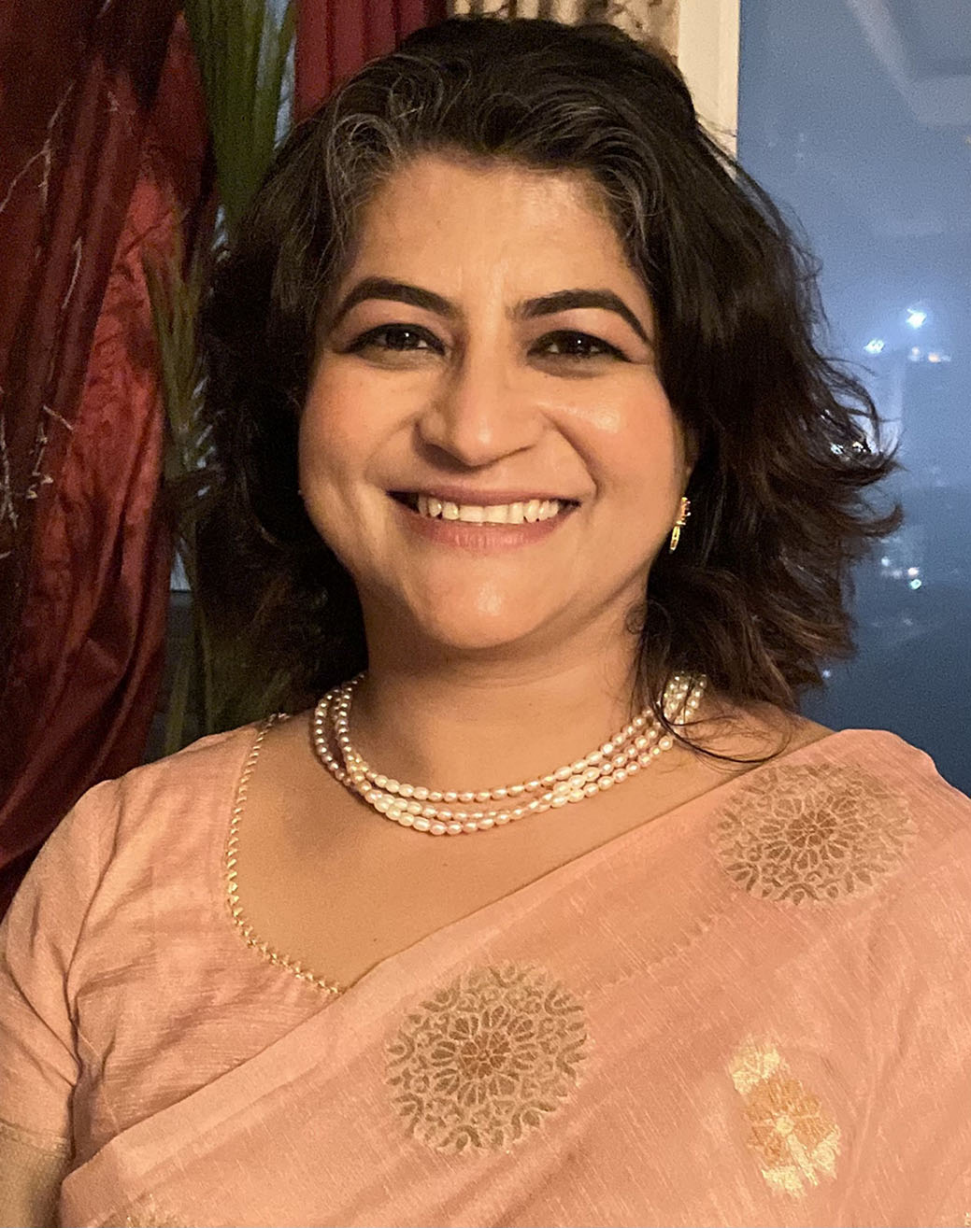


Dr Jyoti Joshi is an antimicrobial resistance (AMR) advisor at the International Centre for Antimicrobial Resistance Solutions (ICARS) in Copenhagen, Denmark. She was awarded her Doctor of Medicine and Specialization in Community Medicine degrees from Lady Hardinge Medical College in New Delhi, India, and a Master’s degree in infectious disease from the London School of Hygiene & Tropical Medicine, UK. Throughout her career, she has been instrumental in researching and implementing public health advances in areas of infectious disease, vaccine safety and AMR. Her work, both directly with communities and in high-level policymaking, is driving significant progress in maintaining and improving public health in several Asian countries and globally. In this interview, Jyoti talks about the need for breaking silos to achieve real progress in AMR control, the importance of family, and how an unexpected career decision can turn out to be the right one.

**After medical school, what motivated you to go into public health?**

Community medicine, which is how public health is typically referred to in India, was a subject at my medical college. That course really inspired me because, unlike treating patients in the clinic, community medicine is about preventing illness by working with people and communities before they become sick. I found this challenging because it is a holistic concept that does not regard health as merely the absence of disease, but it includes wellbeing in all dimensions. I loved clinical medicine, but I loved community medicine even more! After my MD, I worked on reproductive health and human immunodeficiency virus (HIV)/acquired immunodeficiency syndrome (AIDS) prevention in India and then moved to Dubai, where I worked in the Preventive Medicine Department in the government, focusing on vaccines and infection disease surveillance, including the World Health Organization (WHO)'s International Health Regulations. This motivated me to pursue further study, but, due to family commitments, rather than pursuing a full time PhD, I chose a second Master's degree in infectious disease. At that time, colleagues tried to convince me that infectious diseases were decreasing and it would be wiser to focus on non-communicable diseases, but I was intrigued by microbes and recognised that this challenge is ongoing, so I persisted. I'm very happy with my decision and the journey it has taken me on. I now view our efforts for tackling infectious diseases not as a battle, but as a path towards co-existing with microbes around us in a steady state of balance.

“I now view our efforts for tackling infectious diseases not as a battle, but as a path towards co-existing with microbes around us in a steady state of balance.”

**What prompted you to move from infectious disease surveillance to AMR?**

After spending almost 15 years in infectious disease surveillance and vaccines, I realised what a big challenge AMR is to public health. AMR is not traditionally taught in the public health curriculum. Communities are not aware of the problem nor have they been typically involved in addressing AMR. This, in my opinion, is the key reason for the extent of AMR spread and the threats it poses. I'm glad I am working on this issue at the opportune time! The next few years are critical for AMR mitigation, especially in light of COVID-19 [the disease caused by severe acute respiratory syndrome coronavirus 2 (SARS-CoV-2) infection]. AMR is a pandemic that arrived long before COVID-19, and though it has been historically conceptualised as ‘invisible’, the reality is that it pervades our hospitals, communities, farms, rivers and the environment, with very visible impacts on human health. According to a report recently published in The Lancet, an estimated 1.27 million deaths were directly attributed to bacterial AMR in 2019 (Murray et al., 2022).

Today, we are in a position to make the jump from talk to real action, especially within communities, and in addressing it in low- and middle-income countries. We can make visible contributions now, which is what makes work in community medicine so exhilarating. Its beneficial impact affects you both as professionals and as human beings.

**In public/community health, outreach and effective communication with the public are extremely important. Can you tell us about your approach?**

One of my teachers in medical college told me that community medicine is both an art and a science and this stuck with me. The science parts we know how to do, but implementing it among communities requires diplomacy, economic considerations, and sensitivity to behavioural and socio-cultural aspects – that's the art. This aspect requires context and skills. I tried early on to consciously learn this ‘art’ at three levels. First, education in low- and middle-income countries, where I mostly worked, is very siloed. Medicine has its own silo, so do law, engineering, economics, etc. Moreover, experts in different fields do not interact, but they are all needed when working with the community, in community medicine. So when I started my work, I consciously engaged with people that were not in the medical field. This enabled me to leverage greater resources to achieve the best for our communities. Second, I realised early on that how health messaging is packaged and communicated really matters. The more sensitive the topic, like HIV/AIDS used to be, the more important and urgent the communication is. This also involves talking to journalists and local thought leaders, which was not something that we as doctors were taught in medical school. We need to work with journalists, knowing the importance of their deadlines and terms, and communicate effectively through them. Third, understanding the local government structures and systems is essential. As community health specialists, we have vast amounts of technical knowledge. But how this is implemented within the community relies on the existing structures. Public health programmes can fail if they are not scientifically sound or are implemented poorly. Both aspects need to be addressed to ensure the right measures with correct messaging do reach the public in time. A good example of community involvement is the success of the HIV/AIDS prevention programme in involving the affected communities. Although AMR is an even bigger threat, community engagement is lacking. There is, in my view, too much stress on the ‘science’ and not enough on the ‘art’ aspects. We need to change that, and need effective community involvement to succeed.

**Since you mentioned the HIV/AIDS pandemic, what do you think is the key difference in public communication between AIDS, which reached pandemic levels in the 1980s, and COVID-19, which started in the social media era?**

I think tackling HIV/AIDS taught us what works and where the challenges are, and we need to heed those lessons as we attempt to address COVID-19. We also need to be mindful of the simple fact that HIV and SARS-CoV-2 are vastly different and cause completely different kinds of diseases. AIDS is and was a chronic disease, the science to address it progressed relatively slowly and there was (and still is) a strong stigma associated with it. Even though we can now successfully treat HIV-positive people and can efficiently stop the spread with relatively simple measures, we still don't have a vaccine. The situation is the complete opposite in COVID-19 – the acute nature of infection, the airborne transmission and the simple fact that we are still trying to understand the virus and the range of symptoms it causes, including chronic effects, means there still are many grey areas. This makes tackling COVID-19 even more challenging, particularly in the age of social media. With social media nothing gets forgotten, everything is scrutinised, and the anonymity allows people to be brutal. The science about SARS-CoV-2 is constantly evolving and the rapid pace can be complicated for the public to appreciate, unless it is communicated promptly and clearly. This affects the community's ability to embrace the new learnings, adapt through behaviour change and move forward. Timely communication can affect global pandemic mitigation efforts, as was experienced during the 2009 H1N1 influenza and ongoing COVID pandemics, and is unlike the communication required for a slow-evolving chronic disease like AIDS.

**Antibiotics seem to be most commonly discussed, at least in the West, but are there other crucial antimicrobials that are at a high risk of losing their effectiveness?**

AMR indeed encompasses a broad range of agents – antibacterials, antifungals, antiparasitics and antivirals. In low- and middle-income countries in particular, infectious diseases caused by parasites remain a big problem and the need for effective drugs to treat them is high. During severe COVID-19 outbreak in India, mucormycoses, infection caused by a drug-resistant opportunistic fungus, became a significant problem. Similarly, in malaria-endemic regions, artemisinin-resistant malarial parasite species are spreading and we don't have alternative drugs. Extensively drug-resistant (X-DR) tuberculosis and typhoid are fast-emerging problems in Southeast Asia, meaning that currently used antibiotics may soon lose their effectiveness.

**How are the causes and consequences of AMR in these regions different to those in high-income countries?**

I think part of the problem is that antibiotic use in low- and middle-income countries is very different from that in high-income countries. Access to healthcare, including medicines, is a huge challenge, and infectious diseases still have high mortality and morbidity. So, to improve access to care, antibiotics are freely available and are taken even without prescription, in inappropriate doses for incorrect periods, which, together with poor regulatory control, contributes to antibiotic misuse and the burden of AMR. This disproportionately high burden of AMR does not just affect human health, but also other aspects of life, as antibiotics are used in animal farms and then washed out into the environment. Since human health, veterinary medicine, and environment management systems are siloed, fragmented and in different states of maturity in most countries, tackling a ‘One Health’ problem like AMR becomes even more challenging. Antibiotic misuse is the most critical concern, and estimates from the aforementioned study (Murray et al., 2022) have established that AMR is now an even bigger problem than AIDS and malaria combined. Furthermore, the AMR burden is highest in low- and middle-income countries, which unfortunately have the fewest resources to tackle it.

“This disproportionately high burden of AMR does not just affect human health, but also other aspects of life, as antibiotics are used in animal farms and then washed out into the environment. Since human health, veterinary medicine, and environment management systems are siloed, fragmented and in different states of maturity in most countries, tackling a ‘One Health’ problem like AMR becomes even more challenging.”

**Clearly, this is an issue that requires global collaboration. The WHO recently named AMR as one of the top ten global health challenges. What do you think will be the effects of this announcement?**

I feel fortunate to have worked in the field in developing countries and am now based at the newly established International Centre for Antimicrobial Resistance Solutions (ICARS) in Copenhagen, Denmark. ICARS supports low- and middle-income countries to co-create and implement context-specific and cost-effective solutions to tackle AMR. The scale and spread of AMR requires all stakeholders (countries, companies and foundations) to work together to address this challenge. WHO's announcement highlights this imminent threat, but this alone is not enough. Efforts are needed to harmonise standards for the surveillance and reporting of antimicrobial use. The role of the environment in the development and spread of AMR is very poorly understood and understudied, especially in the Global South. It is thus welcome that the WHO, which was already working with the Food and Agriculture Organization (FAO) and the World Organisation for Animal Health (OIE) as part of a tripartite partnership, has now included the United Nations Environmental Programme (UNEP) in a Tripartite Plus platform to tackle AMR. Implementation of all these partnerships at regional, national and sub-national levels in these sectors is, of course, critical.

**You have had a long and successful career. Can you tell us who inspired you along the way?**

There are a several people who have been instrumental in making me continue in my chosen career path. First, my mother, who is no longer alive. She motivated me to study medicine, although I found it really difficult to explain to her that I didn't want to be a physician and would rather work in the community. She respected my decision to specialise in community medicine and was happy as I progressed in my career. I also have a supportive husband, son and mother-in-law, who shared responsibility on the home front as I travelled extensively as part of my job. I have also been inspired by many colleagues (both female and male) who encouraged and helped me deal with balancing career and home life.

**In science, including public health, mentorship is an essential part of training. Can you tell us a bit about your approach to training/mentoring younger colleagues? What, in your personal opinion, are the biggest challenges and rewards?**

I am a team player and value the concept of mentorship. I have always tried to establish a personal connection with each and every team member. I enjoy engaging with people and hearing their thoughts and ideas. I always learn new things from my engagement with colleagues from diverse backgrounds, of any age or gender. I have met some amazing women in my journey so far. I think woman-to-woman mentorship is very important as more and more women in low- and middle-income countries enter the workforce and take on more professional responsibility. As a mentor, I grow with my network, and it fills me with pride to see my own mentees grow and succeed both in their professional and personal lives.

**And finally, what do you enjoy doing outside of work?**

I love spending time with family, especially my teenage daughter who has special needs. She is extremely loving, very demonstrative in her expressions and chatty. She helps me stay grounded and true to my own emotions, enabling me to be mentally strong. I love cooking and trying different cuisines, reading, and spending time with communities in the field.
